# Dynamic characteristics of oxygen consumption

**DOI:** 10.1186/s12938-018-0476-6

**Published:** 2018-04-23

**Authors:** Lin Ye, Ahmadreza Argha, Hairong Yu, Branko G. Celler, Hung T. Nguyen, Steven Su

**Affiliations:** 1 School of Biomedical Engineering, University of Technology Sydney, 15 Broadway, Sydney, Australia; 2 School of Electrical Engineering, University of New South Wales, Sydney, Australia

**Keywords:** Cardiorespiratory response to treadmill exercise, Dynamical modelling, Kernel method, Impulse response identification, Oxygen uptake

## Abstract

**Background:**

Previous studies have indicated that oxygen uptake ($VO_2$) is one of the most accurate indices for assessing the cardiorespiratory response to exercise. In most existing studies, the response of $VO_2$ is often roughly modelled as a first-order system due to the inadequate stimulation and low signal to noise ratio. To overcome this difficulty, this paper proposes a novel nonparametric kernel-based method for the dynamic modelling of $VO_2$ response to provide a more robust estimation.

**Methods:**

Twenty healthy non-athlete participants conducted treadmill exercises with monotonous stimulation (e.g., single step function as input). During the exercise, $VO_2$ was measured and recorded by a popular portable gas analyser ($K4b^2$, COSMED). Based on the recorded data, a kernel-based estimation method was proposed to perform the nonparametric modelling of $VO_2$. For the proposed method, a properly selected kernel can represent the prior modelling information to reduce the dependence of comprehensive stimulations. Furthermore, due to the special elastic net formed by $\mathcal {L}_1$ norm and kernelised $\mathcal {L}_2$ norm, the estimations are smooth and concise. Additionally, the finite impulse response based nonparametric model which estimated by the proposed method can optimally select the order and fit better in terms of goodness-of-fit comparing to classical methods.

**Results:**

Several kernels were introduced for the kernel-based $VO_2$ modelling method. The results clearly indicated that the stable spline (SS) kernel has the best performance for $VO_2$ modelling. Particularly, based on the experimental data from 20 participants, the estimated response from the proposed method with SS kernel was significantly better than the results from the benchmark method [i.e., prediction error method (PEM)] ($76.0\pm 5.72$ vs $71.4\pm 7.24\%$).

**Conclusions:**

The proposed nonparametric modelling method is an effective method for the estimation of the impulse response of *VO*_2_—*Speed* system. Furthermore, the identified average nonparametric model method can dynamically predict $VO_2$ response with acceptable accuracy during treadmill exercise.

## Background

Oxygen uptake ($VO_2$) on-kinetics is an important physiological parameter for the determination of functional health status and muscle energetics during physical exercise [1]. In addition, the $VO_2$ kinetics provides a useful assessment of the body’s ability to support a change in metabolic demand and an insight into the circulatory and metabolic response to exercise. Several studies confirmed that oxygen consumption is mainly controlled by intramuscular factor related metabolic system [2, 3]. Different from heart rate, the oxygen uptake cannot be affected by mood, stress, etc., and is generally considered as the most accurate measurement of the fitness for cardiorespiratory system [4, 5]. The main goal of this paper is to establish a nonparametric model to describe the on-kinetics of the oxygen uptake in response to the speed of treadmill exercise.

Previous researches conducted on the oxygen uptake modelling can be divided into two categories: (i) static status modelling and (ii) dynamic status modelling. For the static status modelling, an early stage study in [6] proposes a linear static model to approximately estimate oxygen uptake for a given range of walking speed. Simple nonlinear static models are also discussed in [7–9] for the compensation of nonlinearities. On the other hand, the transient response of oxygen uptake has captured the interests of many researches. For example, the authors of [10, 11] have developed a first-order system to approximate the process based on step response. Later, the work in [12] has developed a nonlinear dynamic model for oxygen uptake modelling during treadmill exercise with pseudo random binary signal (PRBS) as the input. However, it is relatively difficult for the exercisers to follow the PRBS signal during the treadmill exercise generally.

In real life, the standard deviation of noise in $VO_2$ measurements is quite large due to the limitations of portable gas analyser. For the modelling of a process with large noise, as determining the order is difficult, a nonparametric model such as impulse response (IR) model is a good choice. However, conventional system identification methods for impulse response estimation normally requires relatively complex input such as PRBS [13] to significantly stimulate the system. In previous studies, the response of oxygen uptake can only be roughly modelled as a first-order system due to the lack of suitable modelling techniques. Recently, a new kernel based estimation method has been developed for nonparametric model estimation [14, 15]. To avoid ill-conditioned solutions due to the existence of large noises, a regularised term is incorporated into the cost function [16], which can limit the one-step variation of the estimated parameters. This new kernel based method projects the parameters of IR into a reproducing kernel Hilbert space (RKHS) which can reduce high frequency components in IR model. Furthermore, by using this method, more accurate results can also be obtained enabling us to employ simple inputs such as step input.

In this paper, in order to implement nonparametric modelling of $VO_2$ response to dynamic exercises, the kernel based estimation method has been adopted and modified. An $\mathcal {L}_1$ regularisation term has been added into the cost function to penalise the least significant term of IR which can result in reducing the order of the impulse response model. Particularly, we have demonstrated that this method is still valid when the input of the system is a single step response for this specific* VO*_2_—*Speed* system. For this research, several popular kernels were tested, such as stable spline (SS) kernel, diagonal kernel (DI) and diagonal/correlated (DC) kernel. Furthermore, we showed through several simulation examples that SS and DC kernels can achieve higher accuracy compared to DI kernel for this problem. Eventually, the proposed method was experimentally validated by using the $VO_2$ data collected from 20 participants. The results were compared with the estimated model based on Akaike’s Information Criterion (AIC) selected autoregressive with exogenous terms (ARX) model with predicted error method for parameter estimation.

The main contributions of this work can be summarised as follows. Firstly, a new nonparametric modelling approach has been developed based on the kernel-based impulse response estimation approach, which can efficiently reduce the order of the IR model by incorporating an $\mathcal {L}_1$ penalty term. Secondly, for the developed IR model identification, appropriate kernels selection has been investigated using extensive simulations, and the stable spline kernel (SS) was recommended as the best candidate. Thirdly, it was demonstrated by both experiment and simulation that the proposed method is efficient for the modelling of IR of cardiorespiratory response to dynamic exercise, which often confronts a highly noisy measurement under the stimulation of a simple input signal. Finally, an averaged impulse response model has been established, which is able to quantitatively describe the oxygen update on-kinetics for treadmill exercise.

This paper is organised as follows. In the "[Sec Sec2]" section, the nonparametric method for $VO_2$ modelling is proposed and kernel selection is also discussed. In the "Simulations" section, the simulation is carried out for the validation of the proposed method. In the "Experiments" section, the experimental results are presented. The "Conclusions" section concludes the paper.

## New modelling method for $VO_2$ during exercise

**Fig. 1:**
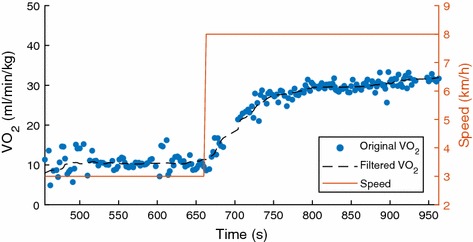
Oxygen uptake during running on treadmill

In most of the previous studies, the step response of oxygen uptake during exercise which is shown in Fig. 1 has been considered as an exponential function [2]:

1 $$\begin{aligned} VO_2(t)=VO_{2}^{0}+{\beta } \left[1-e^{-\frac{{(t-t_d)}}{T_p}}\right], \end{aligned}$$

where $t_d$ is the time delay, $\beta$ is the steady state gain of the system, $T_p$ is the time constant and $VO_2^{0}$ is the baseline value of oxygen uptake. Based on Laplace transform, the transfer function in regarding with Eq. (1) can be derived as follows:

2 $$\begin{aligned} VO_2(s)=\frac{\beta e^{-t_d s}}{{T_p} s+1}. \end{aligned}$$

However, sometimes, a first-order system with time delay cannot lead to the best results due to the pattern of measurements (breath by breath) and the individual variation of oxygen uptake. It is likely that the model of* VO*_2_—*Speed* of some individuals should be described by high-order dynamic systems. However, to correctly identify a high-order system, specific input such as pseudorandom binary sequence (PRBS) is necessary to well stimulate the system. As we know, during treadmill exercise, it is unpractical for users to follow an ideal PRBS signal as input. Therefore, the $VO_2$ uptake during treadmill exercise has been mainly considered as a first-order system and modelled by a first-order ARX model previously. However, a first-order transfer function and a high order transfer function which can be decomposed to serval first-order transfer functions, can lead to quite similar responses with a step input. Therefore, it is generally difficult to identify the correct order for the transfer function which describes the input–output relation. Hence, to overcome this shortage, nonparametric methods are developed to provide better accuracy in this situation. In order to obtain more acceptable results, we exploited a newly developed nonparametric modelling method which make use of finite impulse response (FIR) to describe the system’s characteristic.

### Kernel based estimation method of finite impulse response

In this section, a new kernel based nonparametric estimation method is exploited to model the oxygen uptake during treadmill exercise. For this nonparametric estimation method, it is not necessary to predefine the order of the model in advance. Furthermore, it will be shown that the proposed method can provide stable and smooth estimation comparing to other estimation methods, cf [15].

For nonparametric model, we select *t* with sampling time *T* as the time index. The relationship between the running speed (*u*(*t*)) and oxygen uptake (*y*(*t*)) can be considered as a single input single output (SISO) dynamic system. Therefore, for the impulse response of this SISO system, the discrete time output can be calculated as [17]:

3 $$\begin{aligned} y(t)=G_o(q)u(t)+\varepsilon (t),\quad t=1,2,3\ldots ,M, \end{aligned}$$

where *q* represents the shift operator, i.e. $qu(t)=u(t+1)$, $\varepsilon (t)$ is the Gaussian white noise and $G_0(q)$ is expressed as:

4 $$\begin{aligned} G_o(q)=\sum _{k=1}^{\infty }g_k^0q^{-k}, \quad k=1,2,3\ldots ,\infty , \end{aligned}$$

where $g_k^0$ represents the coefficient from the impulse response $G_o(q)$. For linear response, the impulse response decays exponentially for stable $G_0(q)$. Therefore, normally, the *m*th order finite impulse response is able to describe the system as:

5 $$\begin{aligned} G(q,\varvec{\theta })=\sum _{k=1}^{m}g_kq^{-k},\quad \varvec{\theta }=[g_1,g_2,\ldots ,g_m]^T, \end{aligned}$$

where $\varvec{\theta }\in \mathbb {R}^m$ is the unknown parameter to be identified hereafter. Hence, the model in Eq. (3) can be written as:

6 $$\begin{aligned} y(t)=\varvec{\varphi }^T(t)\varvec{\theta }+\epsilon (t), \end{aligned}$$

where $\varvec{\varphi }(t){\in \mathbb {R}^m}$ contains the input information of the system:

7 $$\begin{aligned} \varvec{\varphi }(t)= \big[u(t-1),u(t-2),\ldots ,u(t-m) \big]^T. \end{aligned}$$

Then, the FIR model can be expressed in matrix form as:

8 $$\begin{aligned} \varvec{Y}_N=\varvec{\phi }_N\varvec{\theta }+\varvec{\varepsilon }_N, \end{aligned}$$

where $N=M-m$, with *M* being the number of collected data points. As an illustration, the *i*th row of $\varvec{Y}_N\in \mathbb {R}^N$, $\varvec{\varepsilon }_N\in \mathbb {R}^N$ and $\varvec{\phi }_N\in \mathbb {R}^{N\times m}$ are $y(m+i)$, $\varepsilon (m+i)$ and $[u(m+i-1), u(m+i-2),\ldots , u(i)]$. Apparently, the straightforward cost function based on Eq. (8) can be expressed as:

9 $$\begin{aligned} {\text {CostFunc1:}\quad \quad \hat{\varvec{\theta }}=\arg } \min _{\varvec{\theta }\in \mathbb {R}^m}||\varvec{Y}_N-\varvec{\phi }_N\varvec{\theta }||^2_2. \end{aligned}$$

By minimising the cost function (9), $\hat{\varvec{\theta }}$ can be derived by least square (LS) estimation or maximum likelihood (ML) estimation easily. However, this is inappropriate for modelling the oxygen uptake impulse response, as the input is only a square signal, and the measurements are normally extremely noisy [12] and thus it is likely that $\varvec{\phi }_N^T\varvec{\phi }_N$ is ill-condition. Hence, to guarantee the validity of the obtained model and avoid any ill-conditioned solution, a regularisation term is crucial to reduce the variation of the estimated parameters in the objective function [16]. Then, the cost function can be considered as:

10 $$\begin{aligned} {\text {CostFunc2:}\quad \quad \hat{\varvec{\theta }}=\arg } \min _{\varvec{\theta }\in \mathbb {R}^m}||\varvec{Y}_N-\varvec{\phi }_N\varvec{\theta }||^2_2+\gamma \varvec{\theta }^T\varvec{W}\varvec{\theta }, \end{aligned}$$

where the first term implies the modelling error, $\gamma$ is a positive coefficient controlling the trade off between the error term and the regularisation term. $\varvec{W}{\in \mathbb {R}^{m\times m}}$ is a weighting matrix, which can be used to prioritise between system parameters. For a normal regulariser $\varvec{\theta }^T\varvec{W}\varvec{\theta }$, regularised least square estimation (ReLS) is a standard method to obtain the solution based on cost function (10). This can be seen as an improved method out of ridge regression or weighted ridge regression [18] depending on the selection of matrix $\varvec{W}$.

However, ReLS cannot achieve desired solution when the input stimulation is insignificant and the measurement has high noise level. In order to obtain a better FIR model of the oxygen uptake model, we introduce a newly developed kernel method based on the work in [14, 15]. Let us recall Eq. (5), assuming that the FIR function $g\in R^{m}$, then the function $g$ in regularisation term can be projected into a reproducing kernel Hilbert space (RKHS), i.e., $g\rightarrow g_{\mathcal {H}}$ ($R^{m}\times R^{m}\rightarrow R^{m\times m}(\mathcal {H})$). The advantage of this transform is the penalisation of the high frequency components in the function *g* [15]. Different from ReLS which only focuses on solving the equalities with ill-conditioned matrices, the regularisation term $g_{\mathcal {H}}$ can perform better for minimising the mean square error of finite impulse response $g(\cdot )$ [17]. If we use the kernel matrix $\varvec{P}$ based on $g_{\mathcal {H}}$ to replace $\varvec{W}$ in Eq. (10), the kernel based estimation method can be expressed as:

11 $$\begin{aligned} {\text {CostFunc3:}\quad \quad \hat{\varvec{\theta }}=\arg } \min _{\varvec{\theta }\in \mathbb {R}^m}||\varvec{Y}_N-\varvec{\phi }_N\varvec{\theta }||^2_2+\gamma \varvec{\theta }^T\varvec{P}^{-1}\varvec{\theta }, \end{aligned}$$

where $\varvec{P}{\in \mathbb {R}^{m\times m}}$ represents the kernel matrix whose details are given in the next section. With the priori information in kernel matrix $\varvec{P}^{-1}$, the estimated $\hat{\varvec{\theta }}$ from Eq. (11) can provide better and smoother results comparing to ReLS or ridge regression [15]. Furthermore, unlike support vector regression (SVR) [19], and for this nonparametric method, the inputs and system parameters from the error term are not projected to a higher dimension as the estimated system parameters from SVR are normally hard to recover from projected space to original system parameters. Furthermore, during the $VO_2$ uptake modelling, this model tends to have relatively large time constant based on the previous research [12]. Hence, we expect that the last several parameters of the estimated FIR approach to zero. Therefore, we need to add an extra $\mathcal {L}_1$ regularisation term as another regulariser to sparsify the transfer function identified, by which the overall cost function can be expressed as:

12 $$\begin{aligned} {\text {CostFunc4:}\quad \quad \hat{\varvec{\theta }}=\arg } \min _{\varvec{\theta }\in \mathbb {R}^m}||\varvec{Y}_N-\varvec{\phi }_N\varvec{\theta }||^2_2+\gamma \varvec{\theta }^T\varvec{P}^{-1}\varvec{\theta }+\alpha ||\varvec{\theta }||_1. \end{aligned}$$

where $\alpha$ is a positive coefficient to control the trade off between $\mathcal {L}_1$ regulariser, kernel regulariser $\gamma \varvec{\theta }^T\varvec{P}^{-1}\varvec{\theta }$ and the error term.

The above equation can be considered as a special case of elastic net [20], in which the $\mathcal {L}_2$ norm regularisation is weighted by kernel matrix $\varvec{P}^{-1}$. Let us rearrange Eq. (12) and define a parameter $\varvec{\phi }^*_N\in \mathbb {R}^{(N+m)\times m}$ as:

13 $$\begin{aligned} \varvec{\phi }^*_N=\frac{1}{\sqrt{1+\gamma }}\begin{bmatrix} \varvec{\phi }_N \\ \sqrt{\gamma }\varvec{R}\end{bmatrix}, \end{aligned}$$

where $\varvec{R}\in \mathbb {R}^{m\times m}$ is the upper triangular matrix from Cholesky factorisation of kernel matrix $\varvec{P}^{-1}$ ($\varvec{P}$ is symmetric).

If denote, $\varvec{Y}^*_N\in \mathbb {R}^{N+m}$ can be defined as:

14 $$\begin{aligned} \varvec{Y}^*_N=\begin{bmatrix} \varvec{Y}_N\\ \varvec{0}\end{bmatrix}. \end{aligned}$$

Then, the cost function (12) can be rewritten as:

15 $$\begin{aligned} {\text {CostFunc5:}\quad \quad \hat{\varvec{\theta }}^*=\arg } \min _{\varvec{\theta }^*\in \mathbb {R}^m}||\varvec{Y}_N^*-\varvec{\phi }_N^*\varvec{\theta }^*||^2_2+\frac{\alpha }{\sqrt{1+\gamma }}||\varvec{\theta }^*||_1, \end{aligned}$$

where $\varvec{\theta }^*{\in \mathbb {R}^m}$ is defined as:

16 $$\begin{aligned} \varvec{\theta }^*=\sqrt{1+\gamma }\varvec{\theta }. \end{aligned}$$

Due to the limitation of the input signal, the input matrix $\varvec{\phi }^T_N\varvec{\phi }_N$ is not orthogonal. Therefore, the close form solution of LASSO [21] cannot be applied to achieve the solution of the optimisation problem of CostFunc5 directly. Here, an interior-point method [22] is adopted for this $\mathcal {L}_1$ norm regularisation for $\hat{\varvec{\theta }}^*$. At the end, $\hat{\varvec{\theta }}^*$ can be restored to $\hat{\varvec{\theta }}$ according to Eq. (16) as:

17 $$\begin{aligned} \hat{\varvec{\theta }}=\frac{1}{\sqrt{1+\gamma }}\hat{\varvec{\theta }}^*. \end{aligned}$$

### Kernel selection

Several kernels have been applied or developed for the proposed nonparametric kernel estimation method, such as polynomial kernel, radial basis function (RBF) kernel, stable spline (SS) kernel, diagonal/correlated (DC) kernel, diagonal (DI) kernel, etc. Due to the use of Cholesky matrix decomposition in the proposed nonparametric modelling method, the kernel matrix must be symmetric positive definite. As a result, SS kernel, DI kernel and DC kernel were selected. SS kernel, DC kernel and DI kernel were developed in [14, 17]. In addition, as the impulse response of a stable process decays exponentially with a certain rate, the SS, DC and DI kernels, which belong to amplitude modulated locally stationary (ALMS) kernel, can often achieve deserved results when identifying FIR model. These three kernels are defined as follows:

- DI kernel:
  18 $$\begin{aligned} P(i,j)=\left\{ \begin{aligned}&c\lambda ^{i},\quad i=j\\&0 \end{aligned}\right. , \end{aligned}$$
  where $$c>0$$, $$1 {>} \lambda {>} 0$$.
- SS kernel:
  19 $$\begin{aligned} P(i,j)=\left\{ \begin{aligned}&c\frac{\lambda ^{2i}}{2}\left( \lambda ^i-\frac{\lambda ^{j}}{3}\right) ,\quad i\ge j\\&c\frac{\lambda ^{2j}}{2}\left( \lambda ^j-\frac{\lambda ^{i}}{3}\right) ,\quad j\ge i \end{aligned}\right. , \end{aligned}$$
  where $$c >0,\lambda >0$$.
- DC kernel:
  20 $$\begin{aligned} P(i,j)=c\rho ^{|i-j|}\lambda ^{|i+j|/2}, \end{aligned}$$
  where $$c> 0$$, $$1>\lambda > 0$$, $$|\rho |\le 1$$ and $$|\rho |\ne 0$$.

More details about the kernels above can be found in [23].

## Simulations

Mostly, the relationship between the oxygen uptake and the jogging speed was considered as a first-order system. To the authors’ best knowledge, due to the individual differences of the body condition of human beings, it is likely that the transfer function model of the $VO_2$ for each person is different in terms of gain value and order. For some people, the relationship between the oxygen uptake and the joggling speed may not be described by a first-order transfer function. Furthermore, it is generally hard to correctly identify the exact order of system through a single input response, especially under large observation noise. The major difference between a first-order system and a high-order system in step response is in their slope and damping. Therefore, it is likely that a second or higher order system is identified as a first-order system through single step response. Therefore, during this “Simulations” section, both first-order systems and second-order systems were considered.

First of all, the performance of the proposed calibration method was tested on the first-order system. In this simulation, we considered a first-order system as

21 $$\begin{aligned} Y(s)=\frac{kX(s)}{{T_p}s+1}, \end{aligned}$$

where time-constant $T_p$ follows the uniform distribution *U*(10, 20) and the gain *k* follows *U*(10, 20). During the simulation, the input signal *X*(*s*) was a step response which jumps from 0 to 1 at time 180 s and remains at 1 for 300 s. For comparison purposes, the estimations of both ARX model and impulse response model were tested. Assuming that the sampling $T_s=1(s)$, the discrete time ARX model of the first-order system in transfer function (21) was assumed as:

22 $$\begin{aligned} y(k)=a_1x(k-1)+b_1y(k-1)+\varepsilon _1,\quad k=2,3,\ldots ,n, \end{aligned}$$

where *n* is the number of samples, $\varepsilon _1$ is the zero mean Gaussian noise with 3*dB* signal-to-noise ratio (SNR). For ARX model, we used the conventional prediction error method (PEM) to solve the unknown parameters $a_1$ and $b_1$. For the estimation of the impulse response model, the proposed method was applied with FIR $m=120$. In paper [24], the authors gave some extra constraints for the parameters’ of kernels: (i) $0.9\le \lambda <1$ for SS kernel; (ii) $0.72\le \lambda <1$, $-\,0.99\le \rho \le 0.99$ for DC kernel; (iii) $0.7\le \lambda <1$ for DI kernel. the settings of kernels are listed as follows after tuning:

- SS kernel: $$c=1$$, $$\lambda =0.98$$
- DC kernel: $$c=1$$, $$\lambda =0.9$$, $$\rho =0.8$$
- DI kernel: $$c=1$$, $$\lambda =0.9$$
- regulariser: $$\gamma =8$$, $$\alpha =10$$

We repeated this simulation for 1000 times, and the fit ratio NRMSE (normalised root mean square error) defined as:

23 $$\begin{aligned} \text {Fit ratio}=\left( 1-\frac{||\hat{\varvec{Y_N}}-\varvec{Y_N}||_2}{||\varvec{Y_N}-\text {mean}(\varvec{Y_N})||_2}\right) . \end{aligned}$$

**Fig. 2:**
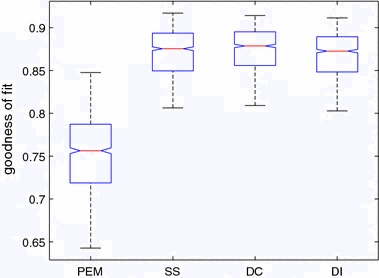
Box plot of fit ratio of estimation from PEM and nonparametric method with SS, DC and DI kernel for first-order model

**Fig. 3:**
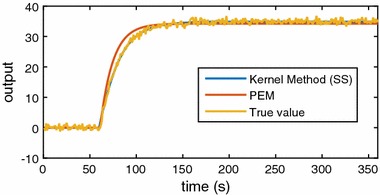
Estimation of one simulation

The results of simulations are reported in Table 1 and the box plot of the results is shown in Fig. 2. From the results of nonparametric method with SS kernel and PEM method, one way analysis of variance (ANOVA) was implemented for the significance test of fit ratio. The p value is less than 0.0001. Therefore, the fit ratio of the kernel estimation method is significantly better than PEM when the noise of measurement is large. Specially, for SS kernel and DC kernel, they can achieve similar results. A simulation result is randomly chosen out of the 1000 simulations and is shown in Fig. 3 to visualise the comparison between the proposed method and the conventional method. Note that to have a clear plot, only the estimated results from the kernel method with SS kernel and PEM are shown. It is evident from this graph that the proposed kernel method can fit better when the measurement is noisy.

**Table 1:** Fit ratio comparison of first-order system simulation

Method	Mean	Standard deviation	Best
PEM	0.7525	0.0444	0.8475
Kernel (SS)	0.8705	0.0264	0.9170
Kernel (DC)	0.8738	0.0254	0.9141
Kernel (DI)	0.8678	0.0254	0.9114

Then, let us consider a second-order system as:

24 $$\begin{aligned} Y(s)=\frac{kX(s)}{({\tau _1}s+1)({\tau _2}s+1)}, \end{aligned}$$

where the variables $\tau _1$ and $\tau _2$ follow *U*(10, 20) and *U*(5, 10), respectively. The gain *k* follows *U*(10, 20). In this simulation, the input signal X(s) is a step response which jumps from 0 to 1 at time 180 s and stays at 1 for 300 s. During the simulation, we used both first-order and second-order model in the ARX model. The second-order ARX model is expressed as:

25 $$\begin{aligned} y(k)= \,& {} a_1x(k-1)+a_2x(k-2) \nonumber \\+ & {} b_1y(k-1)+b_2y(k-2)+\varepsilon _2,\quad k=2,3,\ldots ,n, \end{aligned}$$

where $\varepsilon _2$ is the zero mean Gaussian noise with 3*dB* SNR.

**Fig. 4:**
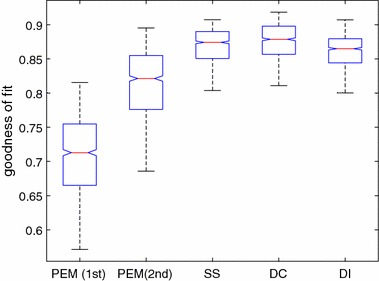
Box plot of fit ratio of estimation from PEM and nonparametric method with SS, DC and DI kernel for second-order model

For impulse response estimation, the SS, DI and DC kernels were selected in the simulation. For the FIR, we set $m=120$, and the settings of the kernels were chosen the same as the first-order simulation. The simulations were repeated for 1000 times, and the results are reported in Table 2 and visualised in the box plot in Fig. 4.

**Table 2:** Fit ratio comparison of second-order system simulation

Method	Mean	Standard deviation	Best
PEM (first-order system)	0.7087	0.0554	0.8157
PEM (second-order system)	0.8133	0.0484	0.8955
SS kernel	0.8694	0.0246	0.9075
DC kernel	0.8758	0.0248	0.9184
DI kernel	0.8615	0.0234	0.9073

**Fig. 5:**
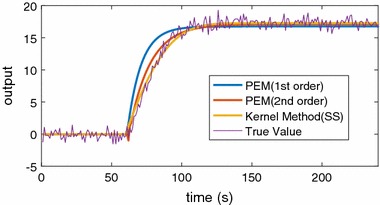
Estimation of one simulation

Based on the results in Table 2, it is clearly seen that the kernel method outperforms previous methods. Another significant advantage is that the order of the model does not need to be determined separately. From the results given in Table 2, if the second-order system is identified as first-order system incorrectly, the differences between the achieved results are significant. Figure 5 shows one randomly chosen simulation. The figure shows that the estimated response from the kernel method fits the best. Although the SS kernel and DC kernel can achieve similar performance so far, paper [17] indicates that SS kernel can outperform DC kernel when the system has higher order. Therefore, we selected SS kernel to estimate the impulse response during our "Experiments" section.

It is also necessary to verify that this method is better than ridge regression. Consider a second-order system as:

26 $$\begin{aligned} Y(s)=\frac{15X(s)}{(10s+1)(15s+1)}. \end{aligned}$$

**Fig. 6:**
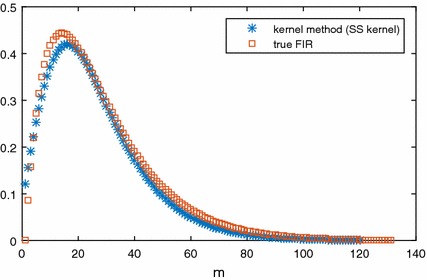
Comparison among true IR and estimated IR based on SS kernel

**Fig. 7:**
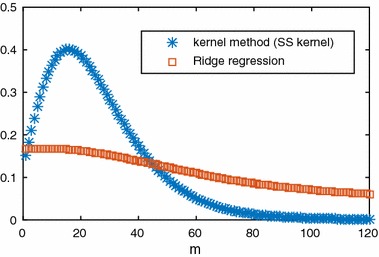
IR from proposed kernel method and Ridge regression

The SS kernel was selected for the proposed method with $c=1$, $\lambda =0.98$ and $m=120$. For ridge regression, whose weighting matrix $\varvec{W}$ in the regularisation term was a simple identity matrix, we also set $m=120$ and both methods share the same weight of regulariser ($\gamma =4$, $\alpha =10$). First, let us visualise the estimated IR from kernel method and true IR in Fig. 6. As we can see from Fig. 6, the estimation output from the SS kernel is very close to the true output without over-fitting. The comparison of results between the kernel method and ridge regression is shown is Fig. 7. From Figs. 6 and 7, it can be seen that the IR model from ridge regression without the kernelised $\mathcal {L}_2$ norm penalty is inaccurate comparing to the kernel method. The results from the kernel method are far better than the classic ridge regression. Specifically, the estimated IR from the kernel method is very close to the true IR.

## Experiments

In order to develop the impulse response model of the dynamic $VO_2$ responses to treadmill exercise, an experimental approach was employed in which 20 healthy males subjects were asked to exercise. The physical characteristics of the participants are shown in Table 3.

**Table 3:** Age and BMI of participants

Subject	Age (year)	Height (m)	Mass (kg)	BMI (kg/m^2^)
Average	38.02	1.77	86.10	27.16
Standard deviation	5.28	0.06	14.05	3.61

All data was acquired by a gas analyser $K4b^2$ (COSMED), which is a portable system for pulmonary gas exchange measurement with true breath-by-breath analysis. The UTS Human Research Ethics Committee (UTS HREC 2009000227) approved this study and an informed consent was obtained from every participant before the commencement of data collection.

Prior to the experiments, all participants were ask to observe the following requirements: including the nutritional intake, physical activity and environment conditions. The participants were instructed to consume a standardised light meal at least 2 h before the experiment. Meanwhile, they were asked not to engage in any other exercises for one day before the experiment. The temperature and humidity of the laboratory were set to 20–25 °C and around $50\%$ relative humidity respectively.

**Fig. 8:**
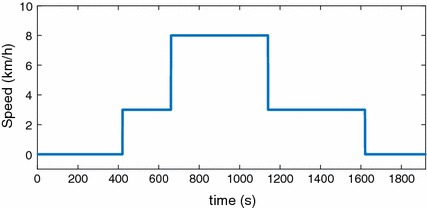
Protocol of exercise for the experiment

**Fig. 9:**
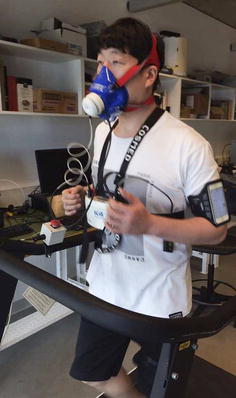
The LabVIEW controlled treadmill system for experiments

During the experiment, each participant was seated for 5 min first, and then stood next to the treadmill for another 2 min. Then, the participants were asked to start walking at 3 km/h for 4 min, followed by a run for 8 min at 8 km/h, and another walk for 8 min and at 3 km/h. At the end, they rested for 5 min. One single experiment took 32 min in total. The protocol of this experiment is shown in Fig. 8. The participants only ran at a relatively low speed (8 min) to avoid anaerobic respiration. The typical experimental scenario with $K4b^2$ gas analyser and the automated treadmill system is shown in Fig. 9.

**Fig. 10:**
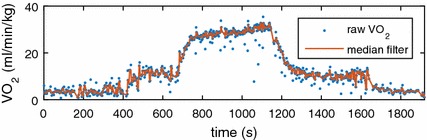
Raw $VO_2$ measurement and filtered measurement of participant 1

In this work, we only focused on the onset $VO_2$ response of treadmill exercise (i.e., from walking to running). Therefore, for the purposes of impulse response modelling, we only took the data from *t*_1_ = 420s to *t*_2_ = 1120s as shown in Fig. 9. Since the data of gas exchange recorded by $K4b^2$ is breath by breath based, the sampling time of $K4b^2$ is irregular, and the quality of the measurements is often relatively low because of the complexity of the gas exchange in cardiopulmonary system. This is also another reason that nonparametric modelling was selected in this study rather than structured modelling. Furthermore, only a 3rd median filter was applied to reduce the measurement noise with minimal influence of breath signals. An experiment was randomly chosen and the result of applying a 3rd median filter is shown in Fig. 10. It is evident from Fig. 10 that the 3rd median filter can efficiently remove the noise, without losing many details from raw signals. As it is mentioned previously, the gas response of $K4b^2$ is breath by breath based, therefore, the sampling time of measurement is not constant. In order to deal with the varying sampling time of $K4b^2$, we used a classic interpolation method [25] to unify the sampling period to 1 s.

Overall, for the estimation of impulse response model, the sampling time was selected as 1*s*, and the order of the model was selected as 300. The IR model can therefore be expressed as a 300 order FIR model:

27 $$\begin{aligned} y[n] &= g[1]u[n-1]+g[2]u[n-2]+\ldots +g[300]u[n-300] \nonumber \\ &=\sum _{i=1}^{300}g[i]u[n-i]. \end{aligned}$$

As we mentioned previously, since this study focused on the onset, we only took the measurements from $t=420$ (starting walking at 3 km/h) to $t=1120$ (nearly finished running at 8 km/h), which is 700 measurements in total. As the order of the FIR is 300, while the walking period only has 240 measurements (4 mins walking), we cloned 200 measurements of walking and inserted it into the selected 700 measurements. Therefore, the data records were extended from 700 to 900. Hence, the input *u*[*i*] is defined as follows, $u[i]=0,\ i=1,2,\ldots ,420$; $u[i]=1,\ i=421,422,\ldots ,900$. With these 900 measurements, firstly, we removed the offset which is the average value of the initial 420 outputs. Then, the proposed nonparametric kernel based estimation was applied to estimate the IR model by using stable spline kernel ($c=1$, $\lambda =0.978$). During the estimation, the coefficients $\gamma$ and $\alpha$ for the $\mathcal {L}_1$ and kernalised $\mathcal {L}_2$ norm were set to 4 and 10 respectively. As a comparison with the conventional method, we also used system identification toolbox from Maltab to estimate the model using the ARX model with AIC for model selection [26]. Then the system was estimated by PEM based on the model that selected by AIC. The fit ratios (NRMSE) were calculated and recorded in Table 4. For a fair comparison, only $\hat{y}$ from the samples 301 to 900 of the selected 900 s samples was compared for both methods as the initial value of $\hat{y}$ of nonparametric method started from 301 s.

**Fig. 11:**
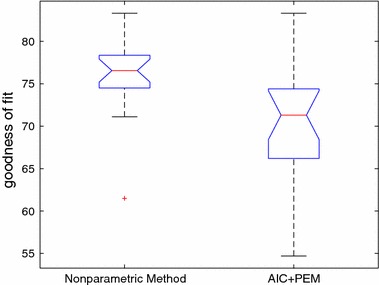
Results comparison for proposed method and classic method

The results are reported in Table 4 and the box plot is given in Fig. 11. From Table 4, it can be seen that the proposed method can significantly outperform the conventional method in most of the cases. It has also higher fit ratio and less standard deviation. Actually, the results in Table 4 show that the proposed method is very effective when the system has high level of noise.

**Table 4:** Goodness of fit

Subject	Nonparametric (%)	AIC + PEM (%)	AIC
Participant 1	83.3	83.3	11
Participant 2	77.0	67.4	7
Participant 3	61.5	54.7	12
Participant 4	75.3	65.4	7
Participant 5	74.7	61.0	4
Participant 6	81.1	73.7	8
Participant 7	81.7	80.1	13
Participant 8	82.2	80.6	11
Participant 9	78.3	74.5	12
Participant 10	72.6	63.6	10
Participant 11	74.3	65.1	9
Participant 12	72.9	71.0	13
Participant 13	71.1	67.0	17
Participant 14	77.8	74.3	15
Participant 15	76.6	74.1	17
Participant 16	76.5	71.6	11
Participant 17	78.0	76.0	11
Participant 18	78.4	72.1	11
Participant 19	76.3	69.4	10
Participant 20	76.4	70.8	13
Average	76.0	71.4	–
Standard deviation	5.72	7.24	–

**Fig. 12:**
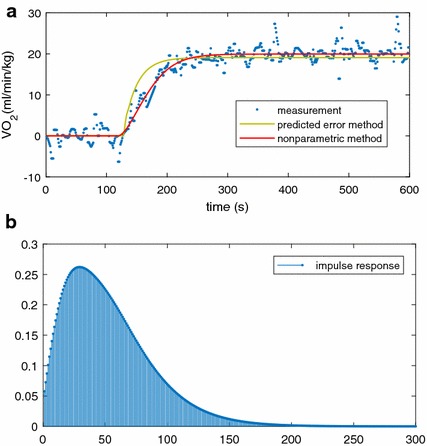
**a** Estimated response for one participant with PEM and nonparametric and **b** the estimated impulse for the participant

Particularly, Fig. 12a shows the estimations based on PEM and the proposed method for one participant. The figure clearly illustrates that the model response of the proposed method fits better to the original measurements, especially for the transient stage.

**Fig. 13:**
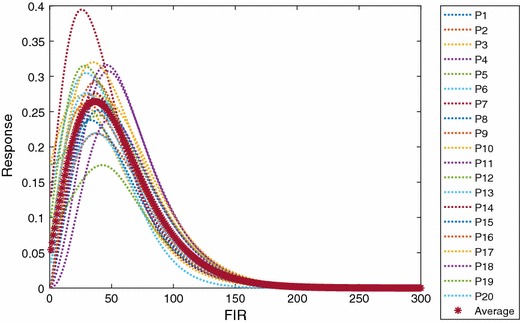
Average IR and individual IR from 20 participants

**Fig. 14:**
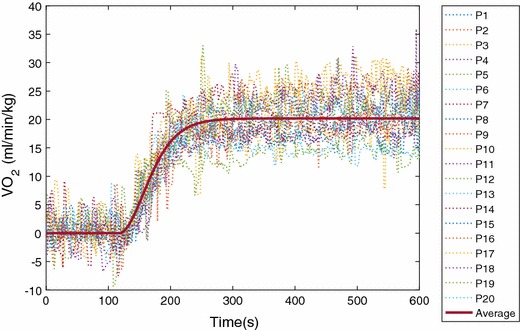
Comparison between estimated $VO_2$ and measurements from 20 participants

Figure 13 shows the estimated impulse responses for all 20 participants. Although the values of the impulse responses are slightly different, the patterns of the responses among participants are similar. This figure also shows that dynamic ${V}O_2$ response to exercise of these 20 participants should be described by a higher order model instead of a simple first-order transfer function as the starting point of the identified impulse response is non-zero. Furthermore, it is evident that the impact of time delay in Eq. (1) is negligible from these results, which is in line with the results reported in [27]. The average impulse response is also shown in Fig. 13. Based on the estimated average impulse response model, we calculated the predicted average $VO_2$ output, and then compared it with the experimental data, shown in Fig. 14. It can be observed that the estimation fits *properly* with the experimental data without over-fitting.

## Conclusions

This paper reports our proposed method for nonparametric modelling of $VO_2$ response to treadmill exercise using a kernel based modelling approach. Several kernel functions have been exploited and tested using different numerical simulations. The stable spline kernel was chosen as it can achieve expected results. With stable spline kernel, the proposed estimation method were tested experimentally using 20 participants. The obtained results showed that the goodness of fit of the proposed method can significantly exceed the prediction error method. We conclude that the kernel based nonparametric modelling method is an effective method for the estimation of the impulse response of the* VO*_2_—*Speed* system. We also believe that the identified FIR model can provide accurate dynamic prediction of $VO_2$ response during treadmill exercise.
